# Prescribing pattern of antibiotics by family physicians in primary health care

**DOI:** 10.1186/s40545-023-00515-6

**Published:** 2023-01-19

**Authors:** Gholamali Karimi, Kourosh Kabir, Babak Farrokhi, Effat Abbaszadeh, Elham Davtalab Esmaeili, Farzad Khodamoradi, Ehsan Sarbazi, Hosein Azizi

**Affiliations:** 1 Student Research Committee, Department of Epidemiology, School of Public Health, Ilam University of Medical Sciences, Ilam, Iran; 2 Savojbolagh Health Center, Alborz University of Medical Sciences, Karaj, Iran; 3 Department of Community Medicine , School of Medicine, Alborz University of Medical Sciences, Karaj, Iran; 4 Executive Deputy National Director for Family Medicine, Health Network Administration Center, Undersecretary for Health Affairs, Ministry of Health, Tehran, Iran; 5 Road Traffic Injury Research Center, Tabriz University of Medical Sciences, Tabriz, Iran; 6 Department of Social Medicine, Faculty of Medicine, Ahvaz Jundishapur University of Medical Sciences, Ahvaz, Iran; 7 Women’s Reproductive Health Research Center, Tabriz University of Medical Sciences, Tabriz, Iran; 8 Department of Epidemiology and Biostatistics, School of Public Health, Tehran University of Medical Sciences, Tehran, Iran

**Keywords:** Antibiotic prescribing, Family physicians, Primary health care, Iran

## Abstract

**Purpose:**

Irrational prescription of antibiotics is an ongoing global public health concern, leading to antibiotic resistance. Understanding the prescribing pattern of antibiotics is important to tackling mal-prescription and antibiotic resistance. We aimed to investigate the pattern and factors affecting outpatients’ antibiotic prescribing by family physicians in Primary Health Care (PHC).

**Methods:**

A cross-sectional study was conducted in 19 PHC facilities in Alborz province. Prescribing pattern of antibiotics was evaluated among 1068 prescriptions by family physicians. Prescribing pattern of antibiotics included prescriptions containing antibiotics, the number of antibiotics per prescription, type, name of antibiotic, and mal-prescription. Multiple logistic regression analysis was used to estimate the adjusted odds ratios and 95% confidence intervals.

**Results:**

Overall, 57% of the prescriptions had ≥ 1 antibiotic and the average number of antibiotics per prescription was 1.27. Amoxicillin was the commonly prescribed antibiotic. There was a significant relationship between age, sex, type of health insurance, work experience of the physician, and seasons with antibiotic prescribing (*P* < 0.05). In 59.31% of antibiotic prescriptions at least one of the scientific criteria was not fulfilled. In the final analysis, after adjusting for the potential confounders, field experts of physicians (OR = 1.59; 95% CI: 1.08–6.17), female sex (OR = 2.23; 95% CI: 1.18–4.21), and winter season (OR = 3.34; 95% CI: 1.26–8.15) were found associated factors with antibiotic prescribing.

**Conclusion:**

The average number of antibiotics per prescription and the percentage of irrational prescriptions were relatively high in this study. There is need to improve antibiotic prescribing patterns among family physicians working in primary health care.

## Background

Irrational use and mal-prescription of antibiotics are a major and ongoing global public health problem, leading to antibiotic resistance both in developing and developed countries that deserve more attention by health systems and policymakers [1, 2, 3]. Rational and appropriate usage of antibiotics were defined as reasonable and appropriate use of antibiotics at an advisable time so that they have had beneficial effects on patients in terms of the virtue of strength, dose and duration of therapy [4]. Rational use of antibiotics depends on pursuing the process of prescription which includes identification of patients’ problem (diagnosis), effective and safe therapy (therapy with drugs or non-drugs options), selecting suitable drugs, dosage, and duration, writing a good prescription, providing enough information to the patient and planning to evaluate treatment responses [5].

Irrational prescribing of antibiotics and the prompt and constant spread of antimicrobial-resistant organisms are major threats to our ability to successfully treat most contagious diseases. In the lack of development of new generations of antibiotic drugs, appropriate use of current antibiotics is required to guarantee the long-term availability of effective therapy for microbial infections [6, 7, 8, 9]. Recently, in a study conducted in Kosova a high and irrational prescription of antibiotics has been reported in primary dental care. A study conducted in four hospitals in southern Ethiopia [10] the percentage of use of antibiotics ranged from 46.7 to 85 and the use of antibiotics in this study was higher than the acceptable range in all the hospitals. Another study evaluated the patterns and appropriateness of antibiotic prescription in outpatients in primary health care settings, China. Out of 3113 antibiotic prescription, 292 irrational prescription were found with inappropriate prescriptions being the most prevalent subtype (79.8%) [11]. In Biswas et al. study [12], the highest prescribed antibiotics were cephalosporins and macrolides in outpatients; and almost 67% prescriptions had complete information on dosage form. A review study indicated that 45% of prescriptions had antibiotics in outpatient settings in Iran [13]. Furthermore, a meta-analysis study in Ethiopia [4] demonstrated that drug prescription patterns and indicators were not fulfilled with the standard values suggested by the WHO.

In Iran, the Primary Health Care (PHC) is the first and nearest line of basic and essential health services which is provided by family physicians and other healthcare providers in the all cities and rural areas. Findings indicated that the irrational prescription of antibiotics and the prevalence of self-medication are leading to antibiotic resistance and side effects in Iran [14, 15]. It is estimated that antibiotics are used as common drugs in Iran and almost half of the patients receive at least one antibiotic during doctor–patient encounters. It is also estimated that more than 8% of hospital admissions in Iran occur due to adverse drug reactions [6, 16, 17]. Accordingly, rational and appropriate antibiotic prescription will decrease antibiotics resistance, adverse drug reactions, toxicity risks, healthcare expenditure, and household costs, and duration of therapy at the global level [17, 18].

Rational prescribing of antibiotic is crucial to tackling antibiotic resistance. Among PHC family physicians, the pattern and the associated factors of antibiotic prescribing are poorly understood in Iran, especially after expanding the family physicians plan to urban areas. We aimed to evaluate family physicians prescribing pattern of antibiotic to outpatients in primary health centers of the Alborz province, Iran.

## Methods

### Study design and setting

A cross-sectional study was conducted in 19 PHC facilities/community health centers from September 2018 to September 2019 in Alborz province, Iran. We evaluated the family physicians' antibiotic prescribing patterns. Prescribing patterns of antibiotics included prescriptions containing antibiotics, the number of antibiotics per prescription, name, form, type of antibiotic, and consumption method, and mal-prescriptions. Family physicians are the chief healthcare providers in the Iranian PHC. The Iranian health system is based on the referral system; the first level is primary level of health services to access all urban and rural populations provided by family physicians and other health service providers. Patients are referred to the second and third levels by family physicians when patients need special health services [19].

**Fig. 1:**
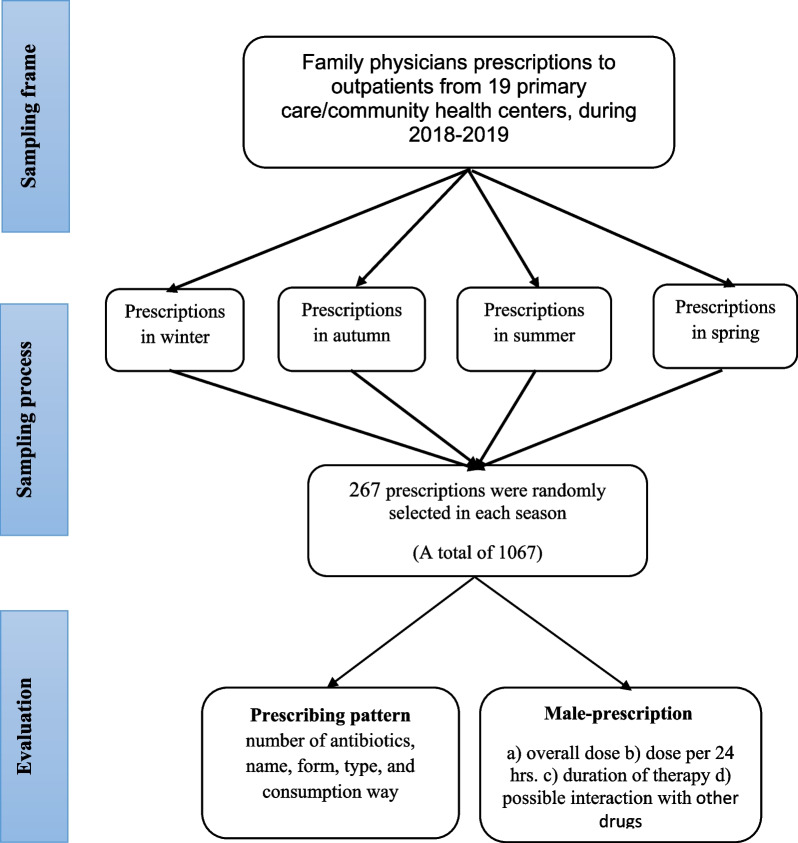
Flowchart for sampling and evaluation process of the family physicians prescription for outpatients

We randomly selected family physicians' prescriptions from County PHC health centers. Given that all family physicians' drug prescriptions (paper) were archived, a total of 1068 prescriptions (267 prescriptions in each season) were randomly selected. All prescriptions were available, therefore in the sampling process, the unreadable or prescriptions with poor information were excluded and the next prescriptions were replaced. More details of the sampling process and the study method are presented in Fig. 1.

The sample size was calculated based on the previous study in Iran (*p* = 0.50) [17] and by considering *α* = 0.05, 95% confidence interval and 20% compensate for poor and ineligible prescriptions and to provide an accurate estimation.

### Data collection

Incorrect or mal-prescription of antibiotics was assessed based on four scientific criteria including a) dose per consumption, b) dose per day, c) duration of therapy and d) possible interaction with other antibiotics or drugs. The prescription was considered incorrect if one or more of the above criteria were not fulfilled. Assessment of the prescriptions was performed by a high expert pharmacist (more than 10 years’ experience) who trained for the study methods and he was not involved in the study or analysis of the outcome measure. The assessment was based on Martindale: The Complete Drug Reference. For this purpose, firstly the symptoms and prescribed antibiotics according to the type of diagnosis (severity of the disease) were extracted from the prescriptions and health records by the clinician and the pharmacist. To do this, after randomly selecting the prescriptions, the patient's medical record in detailed information related to that prescription was also reviewed and evaluated. To this end, the incorrect prescription was evaluated based on the four mentioned standard criteria.

Based on the standard guidelines, each drug and disease has a standard dose per day, duration of therapy, interaction with other drugs or antibiotics in accordance with various ages, genders, weight and disease. The appropriateness of antibiotic prescriptions were assessed regarding the related guideline and the mentioned four criteria, then the incorrect prescriptions were identified for each prescription.

Same number of 276 prescriptions were selected in each season of the year. The dose of all drugs and antibiotics per each consumption, day, duration, possible interaction based on the type of disease stage, and diagnosis were determined by the Complete Drug Reference. The dose of antibiotics (different format) calculated according to milligram (mg) for edible (oral) drugs (such as tablet, capsule) and milliliter (ml) for suspension, ampoule and infusion drugs. The number of drops and the speed of liquid infusion were calculated with this formula: $\text{Numbers of drops (minute)}=\frac{The\,amount\,of\,solution \times drop\,factor}{Time\,of\,infusion (minutes)}.$ The drop factor was special for each drug.

A checklist was used for data collection. In addition to the basic and demographic characteristics of family physicians and outpatients, variables and information such as the name and type of the prescribed antibiotics, form and usage method of drugs, consumption way based on the amount of use for each time, duration of the treatment course, times of use each day, possible interaction with antibiotics or drugs, rate of combination therapy, number and total price of drug items, type of physicians’ graduation (private or government universities), family physicians occupation/position, and the working experience (years) were extracted from prescriptions and medical records. The government medical universities are classified on 3 levels based on the annual scientific report of the Iranian Ministry of Health and Medical Education.

### Data analysis

SPSS software (version 19.0, Chicago, IL, USA) was used for data analysis. The Kolmogorov–Smirnov test was carried out for checking the normality of the variables such as age (*p* = 0.751) and experience of the physicians (*p* = 0.688). Chi-square (*χ*^2^) test was used to compare categorical variables, and the independent *T*-test was used for comparison of normal quantitative variables [20]. Multiple logistic regression analysis was used to estimate the adjusted odds ratios and 95% confidence intervals for the association between antibiotic prescription and the affecting factors. For model building, all independent variables were first assessed with simple logistic regression. Then, all variables with *p*-value less than 0.2 were analyzed with multiple logistic regression using the backward stepwise method [21]. *P*-value < 0.05 was considered significant in all of the tests.

## Results

Table 1 shows the baseline characteristics of the study participants and their association with antibiotic prescribing to outpatients by family physicians in PHC facilities in Alborz province. Overall, 1068 prescriptions by family physicians with 3704 prescribed drugs to outpatients were evaluated. Of the total prescriptions, 607 (56.8%) prescriptions had at least one antibiotic. Moreover, out of all 1068 selected prescriptions, 772 (20.8%) of drugs were antibiotics. It was found a significant difference between antibiotic prescribing and different seasons of the year. Regarding the seasons, 112 (41.9%) prescriptions in the summer and 177 (66.3%) in the winter had at least one antibiotic (*P* < 0.05). Winter season was associated with an increased odds of antibiotic prescribing (OR = 2.36; 95% CI: 1.25–4.68).

**Table 1:** Demographic characteristics and factors affecting antibiotic prescription by family physicians in primary health care, Savojbolagh, Alborz province, Iran

Variables	Prescription (1068)			Crude OR (95% CI)	*p*-value
	With antibiotic *N* = 607	Without antibiotic *N* = 461	Total (%)		
Age of patients					
Mean ± SD 32.4 ± 21.4	26.07 ± 19.3	40.7 ± 21.2	32.4 ± 21.4	4.47 (2.58–8.89)	0.001
Sex of outpatients					
Male	271 (63.3%)	157 (36.7%)	428 (40.0)	1	0.001
Female	336 (52.5%)	304 (47.5%)	640 (60.0)	1.56 (1.22–2.01)	0.001
Sex of physicians					
Male	174 (57.8%)	127 (46.2%)	301 (27.2)	1	1
Female	433 (56.5%)	334 (44.5%)	767 (71.8)	1.6 (0.91–2.38)	0.128
Seasons of year					
Autumn	172 (64.4%)	95 (35.6%)	267 (25.0)	1.51 (1.02–3.54)	0.048
Winter	177 (66.3%)	90 (33.7%)	267 (25.0)	2.36 (1.25–4.68)	0.026
Spring	146 (54.7%)	121 (45.3%)	267 (25.0)	1	1
Summer	112 (42.0%)	155 (58.0%)	267 (25.0)	1.02 (0.78–1.68)	0.421
Type of insurance booklet					
Rural and tribes	216 (50.5%)	212 (49.5%)	428 (40.0)	1	1
Insurance social security	333 (62.4%)	201 (37.6%)	534 (50.0)	1.26 (0.87- 4.65)	0.236
Therapeutic services	46 (55.4%)	37 (44.6%)	83 (7.7)	1.19 (0.76- 6.12)	0.452
Armed forces	12 (52.2%)	11 (47.8%)	23 (2.3)	1.05 (0.82–2.54)	0.760
Employment statues of physicians					
Regular hiring	44 (53.0%)	39 (47.0%)	83 (7.7)	1	1
Contractual hiring	20 (57.0%)	15 (43.0%)	35 (3.3)	1.02 (0.33–1.54)	0.898
Family physician’s contract	410 (57.0%)	310 (43.0%)	720 (67.4)	0.95 (0.70–1.98)	0.553
Plan bill	133 (58.0%)	97 (42.0%)	230 (21.5)	0.88 (0.54–2.13)	0.490
Experience of the physicians					
< 2 years	317 (61.0%)	203 (39.0%)	520 (48.7)	1.38 (1.08–1.77)	0.008
≥ 2 years	290 (53.0%)	258 (47.0%)	548 (51.3)	1	1
Graduation university					
Governmental university	392 (55.3%)	317 (44.7%)	709 (66.4)	0.83 (0.64–1.07)	0.152
Private university	215 (60.0%)	144 (40.0%)	359 (33.6)	1	1
Type of university*					
Type Ι	256 (55.5%)	205 (44.5%)	461 (43.2)	1	1
Type ΙI	118 (53.0%)	105 (47.0%)	223	1.34 (0.83–2.45)	0.330
Type ΙII	18 (72.0%)	7 (28.0%)	25	0.78 (0.76–1.12)	0.188
Type of health center					
Rural	243 (54.7%)	201 (45.3%)	444	1	1
Urban	49 (64.5%)	27 (35.5%)	76	0.86 (0.46–1.39)	0.259
Rural–urban	315 (57.5%)	233 (42.5%)	548	1.90 (0.93–3.10)	0.598

*Scientific rank

Among all evaluated prescriptions, the majority of outpatients 640 (60.0%) were female, while the proportion of received antibiotics was more in male than female (63.3 vs. 52.5%). According to the type of health insurance, outpatients who had social security health insurance booklets had the majority of prescriptions (more than 50%). We found there is a relation between family physicians’ experience and antibiotic prescription. The work experience (≤ 2 years) of family physicians was associated with an increased probability of antibiotic prescription (OR = 1.38; 95% CI: 1.08–1.77). The mean age of outpatients who received antibiotics was 32.4 ± 21.4 years. There was a statistically significant association between patients who had received and no received antibiotics (26.07 ± 19.3) and (40.7 ± 21.2), respectively (Table 1).

**Fig. 2:**
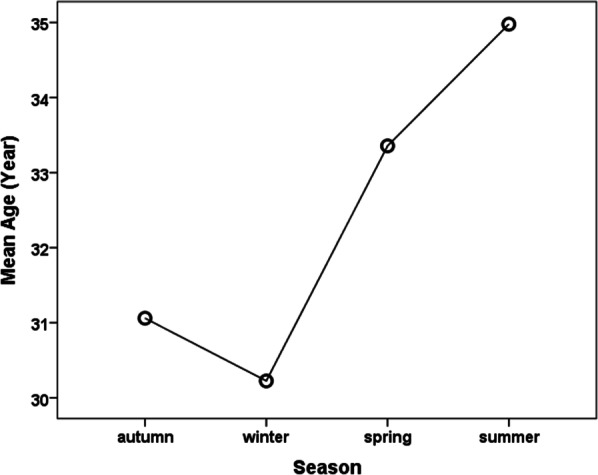
Comparison of the difference in the average of patients’ age based on seasons

Figure 2 shows the age distribution of outpatients by season. The mean age of outpatients was reported as lowest in the winter and highest in the summer.

The average number of drug and antibiotic per prescription was 3.47 ± 1.3 and 1.27 ± 76, respectively. The overall price of prescriptions with antibiotics (37,719,350 Rls.) was higher than prescriptions without antibiotics (25,329,210 Rls.). A relationship was found between price, number of drugs, and prescriptions with antibiotics (Table 2).

**Table 2:** The average and difference mean of number and price of total drug and antibiotic items among prescriptions with and without antibiotic

Variables	Prescription		Mean ± SD	*p*-value	Mean difference	95% Confidence interval	
	With antibiotic	Without antibiotic				Lower bound	Upper bound
Total of drug number	2229	1475	3.47 ± 1.3	0.001	0.47	0.31	0.62
Total of antibiotic number	772	0	1.27 ± 76	–	–	–	–
*Overall price of prescriptions	37,719,35	25,329,210	59,034.23 ± 9161.65	0.018	7196.55	1260	13,132
Price of antibiotics per prescription	21,284	0	35,065.19 ± 4172.86	–	–	–	–

*100 Rls

Table 3 demonstrates the distribution of prescribed antibiotics based on name, the form of drug, and consumption way. Among all prescribed antibiotics, the Amoxicillin capsule 500 mg (10.2%), among drug forms the pill (24.7%), and concerning the consumption method, the oral method (70.59%) had the highest frequency of prescribed antibiotics.

**Table 3:** Frequency of prescribed antibiotics based on name, the form of drug and consumption method by family physicians

Rank	Name	Percentage (%)	Form of drug	Percentage (%)	Method	Percentage (%)
I	Amoxicillin 500 mil.g (cap)	10.23	Tablet	24.7	Oral	70.59
II	Penicillin 1,200,000 u.v (vial)	9.06	Capsule	23.05	Injection	22.27
III	Azithromycin 250 mil.g (cap)	8.03	Suspension	22.7	Local	7.12
IV	Penicillin 6.3.3 u.v (vial)	6.9	Vail	21.3		
V	Cefixime 100(susp)	5.8	Ointment	4.2		
VI	Cefixime 400 mil.g (tab)	5.6	Drop	2.7		
VII	Co-amoxiclav 312 (susp)	5.5	Ampoule	0.9		
VIII	Other antibiotics	48.88				

Table 4 indicates the proportion of incorrect prescriptions (with and without antibiotics) among 1068 prescriptions by the family physicians. The common mal-prescription was criterion (a); incorrect dose per day; and the proportion of mal-prescription in criterion (a) was 34% and 38.7% in prescriptions with and without antibiotics, respectively.

**Table 4:** Distribution of incorrect prescriptions with and without antibiotics by family physicians in primary health care

Type of incorrect prescription	With antibiotic prescription (%)	Without antibiotic prescription (%)	Total incorrect prescriptions (%)
Dose per consumption	10.23	12.52	21.9
Doses per day	34.06	38.71	67.72
Duration of treatment	15.02	17.13	29.97
Interaction with other antibiotics	23.22	17.	40.63

Table 5 shows multiple logistic regression analysis results for the association between antibiotic prescribing and effective factors after adjusting for the potential confounders. Family physician field experience (time since graduation) (AOR = 2.23, 95% CI: 1.18–4.21), female sex (AOR = 1.59, 95% CI: 1.086–6.17), and winter season (AOR = 3.34, 95% CI: 1.26–8.15) were associated with antibiotic prescribing.

**Table 5:** Multiple logistic regression results on factors associated with antibiotic prescribing by family physicians

Variable	Adjusted* OR (95% CI)	*P*-value
Sex of family physician		
Male	1	1
Female	1.59 (1.08–6.17)	0.037
Experience of the physicians		
< 2 years	2.23 (1.18–4.21)	0.013
≥ 2 years	1	1
Season of prescribing		
Spring	1	1
Summer	0.49 (0.02–1.08)	0.056
Autumn	1.08 (0.05–2.30)	0.241
Winter	3.34 (1.26–8.15)	0.027

Adjusted for age of patients, and physicians, type, and level of the university

## Discussion

This study evaluated the pattern of antibiotic prescribing for outpatients and it’s affecting factors among family physicians in the Iranian PHC system. Family physicians are the most important and the major healthcare providers in PHC facilities in Iran. Rational prescribing of antibiotics among family physicians can provide a significant role to reduce antibiotic resistance and health expenditures in Iran and global health systems. Furthermore, previous studies had examined mostly the prescribing of antibiotics by general practitioners, specialists, or dentists [22, 23]. This study is one of the few studies that focused on family physicians of community health center of PHC setting in Iran.

The proportion of prescriptions with antibiotics in this study (56.8%) is similar to the result of an Indian study that reported 55%, [24] but this proportion was reported 45% in a study conducted in Sabzevar, Iran [25]. Our findings showed that a high proportion of antibiotic prescription in this study. The reasons for this high and irrational prescription of antibiotics by family physicians may include beliefs, different social and cultural factors among patients and physicians, high rate of environmental pollutants in accordance to the industrial zone, especially the air pollution, due to neighboring the capital city of Tehran and the Karaj metropolis and being suspicious to infectious diseases such as sinusitis and pharyngitis. Likewise, it may occur due to easy access to medications and drugs and the low price of drugs in comparison to other countries.

The present study indicated that the high field experience of family physicians, sex, and seasons are the most important factors associated with antibiotic prescribing in this County. Similar results were found by Safaeian et al. in Isfahan Province of Iran [17]. Yet, the rate of antibiotic prescription in our study was less than a study conducted in Tehran metropolis (62.39%) [26], which supports the effect of the accessibility factor and the role of environmental pollutants that lead to more infections. Tehran, the Capital of Iran, is a highly crowded city with high air pollution at the global level. In Tehran, it is easy to access prescribed antibiotics by family physicians. The low work experience of family physicians (newly graduated), the high proportion of specialists (almost 70% of all specialists in the country), self-medication, and the habit and cultural factors of the people altogether encourage them to refer to physicians who prescribe many antibiotics, particularly in injected forms.

The average rate of prescribed drug number in this study was 3.47 per prescription, this finding is almost in agreement with the Iranian protocol of family physicians' average prescriptions which is 3.5 number of drugs [27]. However, this portion is higher than in developing countries which is between 2.2 and 3.8, and in developed countries about 1.3 to 2.2 [28, 29]. There is need to improve the level of knowledge and skill of antibiotic prescribing patterns in family physicians working in primary health care which was also highlighted by Sami et al. study in Iran [30].

Another related factor to the antibiotic prescribing in the present study was the impact of the seasons so that the number of antibiotic prescriptions increased as the cold season came. The mean age of outpatients in winter was lower than warm seasons in this study. This finding is in agreement with national studies [17, 31]. It may be that the majority of outpatients in winter are children and they are susceptible to respiratory infections and flu while the proportion of advanced-age outpatients is higher in summer.

One of the most important criteria for evaluating the correct and rational prescribing of antibiotics is compliance with international valid guidelines and up-to-date medical science. Our findings showed that the majority of the prescribed antibiotics did not follow the correct scientific method in our study area. This defect may be a major determinant of the development of antibiotic resistance and may be a threat to human life.

In this study, incorrect and unscientific antibiotic prescriptions was assessed based on four criteria including antibiotic dosage per consumption, daily doses, duration of therapy and interaction with other antibiotics or drugs. Our findings showed that the majority of antibiotic prescriptions by family physicians no fulfilled four correct criteria. This finding has been observed in other studies in Iran and other countries [32–36] while this issue was reported as slightly high in our study. Therefore, reducing the number of antibiotics in each prescription and inappropriate and irrational prescribing of antibiotics are major concerns for drug resistance and it is a challenge for the country's health system that deserves prompt attention to improve and modify it.

In the present study, injection form of antibiotics was prescribed more than other forms with 22.27%. Although this amount was reported at 49% in a study in Urmia city of Iran [37] which is higher than our study, the injection form was reported less than our study in the Bhopal zone of India with 13.8% [38]. Therefore, a high percentage of antibiotics (injection form) are prescribed by family physicians shows an irregular form of injecting antibiotics by them. It may have resulted from cultural-social factors and belief in the high effect of injection form of drugs by the patients which, besides being expensive regarding the oral form, sometimes are dangerous for patients [39]. Another reason may be the existence of ampoule injecting room in most of therapeutic and health centers and following the desire of patients to receive whole services from the place of their refer [17, 26]. Amoxicillin and Penicillin were the most commonly prescribed drug in our study and also a study by Dong et al. in China [40].

The price of antibiotics in proportion to the total price of drugs of each prescription was 33.7% and for the prescriptions that included antibiotics was 56.4%. This is a high number in comparison to the results of the study from Urmia, Iran with 35% [37] and the findings from other countries such as France with 34.7% and the USA with 33% [41, 42]. The high rate of antibiotic prescription imposes more economic burden on families and the health system of the country. A previous study stated that up to $3500 can be saved by limiting antibiotic prescribing [43]. Nevertheless, the average price of prescriptions in this study which was 59,034 RIs in comparison to the U.S, which was $75 in 2006, was a low number that shows the lower price of the drugs and their unreal cost in the country [44].

## Limitations

In this study, we evaluated the pattern of family physicians' antibiotic prescribing to outpatients in PHC system of Iran. Although we carried out multiple logistic regression analysis to estimate adjusted ORs in the association between antibiotic prescribing and related factors, it may be affected by many contextual, educational, cultural and qualitative variables [45] other than the variables and factors assessed in this study.

## Conclusions

Our findings indicated that family physicians' antibiotic prescribing is relatively high. Nonetheless, not only the average number of antibiotics per prescription is high, but also incorrect prescribing of antibiotics needs to be adjusted. The findings of this study can be useful in tackling the pattern of irrational and mal-prescribing of antibiotics in Iranian PHC by family physicians. There is a need to improve the level of knowledge and skill of antibiotic prescribing patterns among family physicians working in primary health care.

## Recommendations

Qualitative and holistic studies are suggested for a better understanding and reasons for the high pattern of antibiotic prescribing by family physicians.

The study highlights the need for high-quality, and evidence-based interventions to improve rational antibiotic prescribing by family physicians. This study also showed the need for coping strategies that may assist to decrease antibiotic mal-prescription.

## Data Availability

The datasets generated and/or analyzed during the current study are available from the corresponding author on reasonable request.
